# Incorporation of functionalized gold nanoparticles into nanofibers for enhanced attachment and differentiation of mammalian cells

**DOI:** 10.1186/1477-3155-10-23

**Published:** 2012-06-11

**Authors:** Dongju Jung, Itsunari Minami, Sahishnu Patel, Jonghwan Lee, Bin Jiang, Qinghua Yuan, Liu Li, Sachiko Kobayashi, Yong Chen, Ki-Bum Lee, Norio Nakatsuji

**Affiliations:** 1 Institute for Integrated Cell-Material Sciences (WPI-iCeMS), Kyoto University, Kyoto, 606-8501, Japan; 2 École Normale Supérieure, CNRS-ENS-UPMC UMR 8640, 24 rue Lhomond, Paris, 75005, France; 3 Department of Chemistry and Chemical Biology, Rutgers, The State University of New Jersey, Piscataway, NJ, 08854, USA

**Keywords:** Molecule-doped nanofiber, Synthetic nanofiber substrata, Functionalized gold nanoparticles

## Abstract

**Background:**

Electrospun nanofibers have been widely used as substrata for mammalian cell culture owing to their structural similarity to natural extracellular matrices. Structurally consistent electrospun nanofibers can be produced with synthetic polymers but require chemical modification to graft cell-adhesive molecules to make the nanofibers functional. Development of a facile method of grafting functional molecules on the nanofibers will contribute to the production of diverse cell type-specific nanofiber substrata.

**Results:**

Small molecules, peptides, and functionalized gold nanoparticles were successfully incorporated with polymethylglutarimide (PMGI) nanofibers through electrospinning. The PMGI nanofibers functionalized by the grafted AuNPs, which were labeled with cell-adhesive peptides, enhanced HeLa cell attachment and potentiated cardiomyocyte differentiation of human pluripotent stem cells.

**Conclusions:**

PMGI nanofibers can be functionalized simply by co-electrospinning with the grafting materials. In addition, grafting functionalized AuNPs enable high-density localization of the cell-adhesive peptides on the nanofiber. The results of the present study suggest that more cell type-specific synthetic substrata can be fabricated with molecule-doped nanofibers, in which diverse functional molecules are grafted alone or in combination with other molecules at different concentrations.

## Background

Although the invention of electrospinning was disclosed in the 1930s, electrospinning-related studies have exponentially increased in the last few decades owing to the demands of nanofibrous structures for diverse applications [1]. In particular, electrospun nanofibers became popular in tissue engineering as a substratum because of their structural similarity with collagen fibers in natural extracelluar matrices [2,3], which are composed of submicrometer-sized collagen fibers [4], proteoglycans, and basal membranes [5]. Many biocompatible natural polymers, synthetic polymers, or their copolymers have been successfully used to produce electrospun nanofibers for mammalian cell culture. The components of natural polymers are usually structural proteins identified in extracellular matrix and their derivatives, such as collagen, laminin, and gelatin, whereas poly-ϵ-caprolactone, poly-L-lactic acid, and poly-D,L-lactic-co-glycolic acid are synthetic polymers commonly used to produce biocompatible electrospun nanofibers [6-10]. The natural polymers have strong affinities to mammalian cells, but they have structural inconsistency in wet conditions and large variations depending on their origin, subtype, and concentration. Conversely, synthetic polymers produce structurally consistent nanofibers that lack affinity for mammalian cells. Thus, achieving the affinity of the natural polymer and the structural consistency of the synthetic polymer is desired to produce functionally strong and structurally consistent nanofibers [11,12]. Moreover, the development of functional peptides that mimic the affinity of full-length proteins has enabled high-density localization of the cell affinity function on the synthetic nanofibers [13,14]. Among the developed peptides, a peptide composed of 3 amino acids, arginine-glycine-aspartic acid (RGD), might be the most widely used grafting material to improve cell affinity for the synthetic substrata [15-17]. The tripeptide RGD sequence is commonly identified among proteins that constitute an extracellular matrix, such as laminin, fibronectin, and vitronectin, to which integrin receptors bind [18]. Despite its short sequence, the RGD peptide has been known to mimic the affinity of full-length proteins for integrin receptors [19]. Moreover, cyclic analogues of the RGD peptide that have higher affinity for mammalian cells than their linear counterpart have been developed [20,21]. Besides the RGD peptide, heparin-binding peptides (HBP) that bind to the anionic heparin polysaccharide, which is a component of extracellular matrix, have been demonstrated to potentiate adhesion, locomotion, and growth of mammalian cells, including human pluripotent stem cells (PSCs) [22,23]. Coadministration of the 2 peptides, the RGD peptide and HBP, on the synthetic substratum facilitated long-term culture of human PSCs [23].

PSCs attract a lot of attention for their potential to supply any kind of somatic cells in the body. In addition to embryonic stem cells (ESCs), which are mainly produced from a preimplantation embryo [24-26], the generation of induced PSCs (iPSCs) [27,28] has gained even more attention because iPSCs could be generated from somatic cells. In particular, patient-derived iPSCs have great potential for cell therapy and development of patient-specific diagnostics and drugs. Using human PSCs to differentiate cardiomyocytes is a good model to explore such potential of PSCs because cardiomyocytes can be used for cell transplantation, screening small molecules that modulate contractility of the heart, and evaluating efficacy of drugs for heart diseases. To exploit the full potential of cardiomyocytes, it is necessary to develop an optimized substratum that potentiates generation of contractile cardiomyocyte colonies. Conventionally, a gelatin-coated plate has been used, but recent findings indicate that laminins better potentiate cardiomyocyte differentiation [29,30], which is consistent with the high expression of laminins in mammalian heart [31]. Laminin is a class of glycoproteins composed of α, β, and γ chains, from which 15 different laminins are produced in human tissues [32]. Among the laminins, laminin-511 (composed of α5, β1, and γ1 chains) and laminin-211 (composed of α2, β1, and γ1 chains) have proved to be natural protein substrata that facilitated the maintenance of human and mouse PSCs for a longer period in vitro [33,34].

Herein, we examined the potentiating activity of a functionalized nanofiber substratum for cardiomyocyte differentiation in comparison with that of laminin-211. The PMGI nanofiber was selected to be functionalized through electrospinning owing to its proven rigidity that enables incorporation of small fluorescent molecules through co-electrospinning [35], similar to the incorporation of fluorescent proteins in polyurethane nanofibers [36]. It is intriguing that the small fluorescent molecules and proteins maintained their fluorescence in the nanofibers even though high voltage was applied during the electrospinning processes, suggesting that adhesive peptides can be grafted onto nanofibers through co-electrospinning and still maintain their adhesive function. We confirmed this hypothesis by potentiating cardiomyocyte differentiation of human PSCs with the adhesive peptide-doped nanofiber substratum.

## Methods

### Electrospinning and fabrication of nanofiber substrata

Electrospinning was processed with 13 % (*w*/*v*) PMGI polymer solution (Microchem, Newton, MA) as described previously [35]. Briefly, the concentrated solutions of functional molecules were added to the PMGI solution up to 10 % (*v/v*) and mixed completely: concentrated stock solutions were 10 mM fluorescent molecules such as fluorescein and porphine dissolved in ethanol, 1 mM fluorescent peptides dissolved in dimethylsulfoxide (DMSO), and 10.7 nM peptide-labeled AuNPs dissolved in water. The mixed solution was loaded into a syringe equipped with a 21 one-fourth-gauge blunt-ended steel needle (Nipro, Osaka, Japan). To produce nanofibers, 8 kV was applied between the needle and a grounded collector, which was a silicon wafer covered by aluminum foil, separated 10 cm apart, while the PMGI solution was released continuously out of the syringe at a speed of 0.8 μL/min via a syringe pump. The diameters of the collected nanofibers ranged from 300 to 500 nm. Synthetic nanofiber substrata were fabricated by repeated pipeting of the collected nanofibers, which were fully soaked in autoclaved Milli-Q water (Millipore, Billerica, MA), against the surface of the polystyrene dishes. Approximately, nanofibers produced with 4 μL of PMGI solution were used to coat a 35 mm dish. The unbound nanofibers were washed out with autoclaved Milli-Q water. The substratum was sterilized under UV light for 4 h before use.

### Testing the release of fluorescent molecules

Each fluorescent molecule, the sodium salt of fluorescein (fluorescein) and the 5,10,15,20-Tetra(4-pyridyl)-21 *H*,23 *H*-porphine (porphine, Sigma-Aldrich, St. Louis, MO), was dissolved in DMSO at a 10-mM concentration and added to the 13 % PMGI solution at a 10 % (*v/v)* ratio. The fluorescent molecule-doped nanofibers were used to construct nanofiber substrata using 10-cm polystyrene dishes. Each substratum was incubated at 37°C in 10-mL phosphate-buffered saline (PBS). Every 5 d, the residual fluorescence intensities of the nanofiber substrata were measured using a fluorescent microscope (IX71; Olympus, Tokyo, Japan) and the accompanying MetaMorph image analysis software (Molecular Devices, Sunnyvale, CA). The size of the measured area, exposure time, and threshold were preset for the equal measurement of the fluorescence intensities of 5 different areas in each substratum. For the peptide-releasing test, 3 peptides were custom synthesized (Invitrogen, Tokyo, Japan): a hydrophobic peptide composed of 6 leucines, a negatively charged peptide composed of 6 glutamic acids, and a positively charged peptide composed of 6 lysines. To monitor their release, a lysine labeled with fluorescein isothiocyanate (FITC) was added to each of the peptides. The FITC intensities in the nanofiber substrata were measured as described for the small fluorescent molecules, with which the amounts of the residual peptides in the nanofiber substrata were quantified.

### Transmission electron microscopy and fast Fourier transform analysis

Solutions of 20-nM AuNP were used to dope the nanofibers through electrospinning. The AuNP-doped nanofibers were fully soaked in Milli-Q water and fragmented by pipeting. For transmission electron microscopy (TEM) observation, microdroplets of the fragmented nanofibers or the AuNPs were deposited and dried on a plastic holey film covering a copper grid. TEM was done using a JEM-2200FS (JEOL Ltd., Tokyo, Japan), operating at 200 kV. The fast Fourier transform (FFT) analysis was done with the DigitalMicrograph software package (Gatan Inc., Pleasanton, CA).

### Scanning electron microscopy

Mouse R1 ESCs were spread over unfixed PMGI nanofiber mesh and cultured for 1 week under growth medium, which was composed of DMEM-F12 supplemented with 15 % fetal bovine serum, 0.1 mM 2-mercaptoethanol, non-essential amino acids, and 1,000 U/ml mouse leukemia inhibitory factor (LIF; ESGRO) from Millipore (Billerica, MA). The cells were fixed using 1 % glutaraldehyde solution in PBS for 1 h, and then soaked in 100 % *t*-butyl alcohol for 1 h. After washing with PBS, the cells were dried at 4 °C for 30 min. Then, the cells were covered with a 5-nm-thick gold layer through a sputtering at 200A for 15 sec. Samples were observed with a microscope (JCM-5000; JEOL Ltd., Tokyo, Japan). Using the same method, plain PMGI nanofibers were prepared for structural analysis with the SEM microscope.

### AuNP conjugation with peptides

The unconjugated AuNPs (15 nm; Ted Pella, Redding, CA) were labeled with functional peptides using a 2-step method. First, a 1-mM mixture of 16-mercapto-hexadecanoic acid (MHDA; Sigma-Aldrich, MO) and the polyethylene glycol (PEG)-based molecule, which was dissolved in ethanol at a 1:3 ratio (MHDA/PEG), was added to a basic AuNP solution (pH 11, NaOH) and stirred for 24 h. In most cases, the mole fraction of the thiols in the solution was similar to the mole fraction of the thiols bound to the nanoparticles. The solution was then filtered 3 times with a 10,000-MWCO filter (Millipore, Billerica, MA) by adding Milli-Q water at each step. The second step involved linking the peptides to the AuNPs via 1-ethyl-3-(3-dimethylaminopropyl)carbodiimide (EDC; Sigma-Aldrich, MO) and *N*-hydroxysuccinimide (NHS; Acros Organics, Geel, Belgium). To the AuNP solution being stirred at room temperature, we added 0.3-mM EDC and 0.75-mM NHS, stirred the solution for 45 min to activate the carboxyl group, and then filtered the solution 3 times with 25-mM 2-(*N*-morpholino)ethanesulfonic acid (MES) buffer. A solution containing a 10-M excess of a mixture of cRGD peptide (Peptide International, Louisville, KY) and/or heparin-binding peptide I (BioVision, Milpitas, CA) was added dropwise to the AuNP solution, which was preadjusted to pH 7.5 with 100-mM 4-(2-hydroxyethyl)-1-piperazineethanesulfonic acid (HEPES) buffer, and stirred for 2 h. The AuNP solution was filtered 3 times using a 10,000-MWCO filter to remove the unreacted molecules and finally reconstituted with Milli-Q water. Each step of the labeling was confirmed by measuring the size of the AuNPs using a Zetasizer (Nano-ZS90; Malvern Instruments Ltd., Malvern, UK).

### Contact angle measurement

Nanofiber meshes (1-mm thickness) were laid on a solid surface on which a water drop was placed. Static contact angles of the water drops were measured using the sessile drop method on a homemade contact angle instrument and the Low Bond Axisymmetric Drop Shape Analysis plugin [37] for ImageJ 1.440 software.

### HeLa cell adhesion

The nanofiber substrata were constructed with the PMGI nanofibers containing plain AuNPs, FLAG and PEG-labeled AuNPs, or cRGD and PEG-labeled AuNPs. Over the nanofiber substrata, the same number of HeLa cells (500 cells/cm^2^) were seeded and incubated at 37°C in the presence of 5 % CO_2_ for 6 h. HeLa cell attachment was monitored using a microscope, and the bound cells were quantified by counting the detached cells after trypsinization from the 3 dishes of each nanofiber substratum.

### Cardiomyocyte differentiation of human PSCs

A human ESC line (KhES-3) and a human iPSC line (IMR 90-1) were maintained on mitomycin C-treated mouse embryonic fibroblasts (MEFs) with primate ESC culture medium (ReproCELL, Yokohama, Japan) containing 5 ng/mL basic fibroblast growth factor (Wako, Osaka, Japan). The human ESC line was used in conformity with the guidelines for derivation and use of human embryonic stem cells of the Ministry of Education, Culture, Sports, Science, and Technology (MEXT) of Japan. Cardiac differentiation was carried out as described in a previous study [38], with minor modifications. Briefly, human PSCs were cultured in suspension for 24 h in ultra-low-attachment dishes (Corning, Lowell, MA) to form aggregates and then transferred onto 3.5-cm polystyrene dishes coated with the nanofibers or 20-μg/mL human recombinant laminin-211 (BioLamina, Stockholm, Sweden). The generation of cardiac colonies was enhanced by adding WNT signaling inhibitors, which were identified by library screening (Minami et al., in preparation), for days 3–9 of cardiac differentiation. Beating cardiac colonies were counted on day 10; cell clumps showing synchronized beating were regarded as a single colony, irrespective of their size.

## Results

### Release of the fluorescent molecules

**Figure 1:**
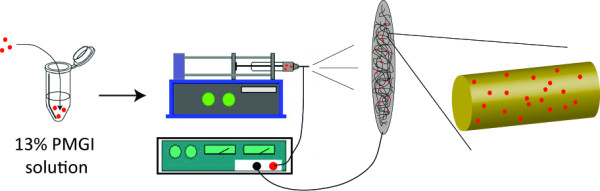
**Construction of molecule-doped nanofibers through electrospinning.** Small molecules, peptides, or gold nanoparticles were premixed in the 13 % PMGI solution with which electrospun nanofibers were produced. The premixed molecules, indicated with red dots, were integrated onto the nanofiber during the electrospinning process.

**Figure 2:**
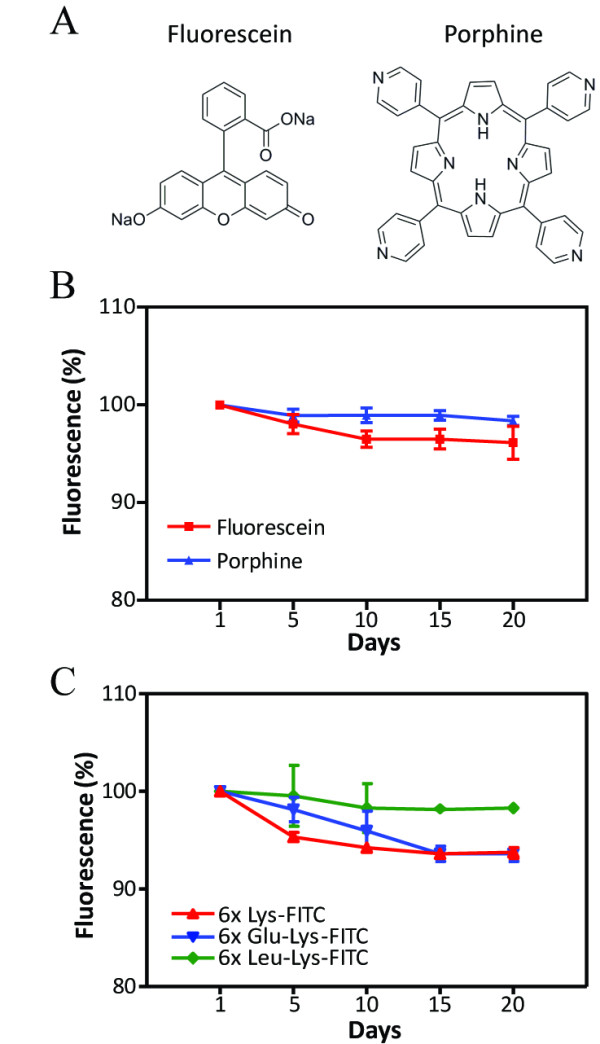
**Release of small fluorescent molecules and peptides from the PMGI nanofibers.** The 2 fluorescent small molecules, fluorescein and porphine (**a**), and the 3 peptides (6X Lys, 6X Glu, and 6X Leu) containing FITC-labeled lysine at each carboxy terminus were used to dope the nanofibers with which polystyrene dishes were coated. The dishes were incubated at 37°C in PBS. The residual amounts of the fluorescent small molecules (**b**) or fluorescent peptides (**c**) in the nanofibers were quantified using a fluorescent microscope. The fluorescence intensity of each nanofiber substratum on the first day was defined as 100 %. A.U. indicates arbitrary unit. Error bars: SEM, n = 5.

Generally, molecule-doped nanofibers have been developed for controlled release of the doped molecules [39-41]. In contrast to this, nanofibers used as a substratum should retain the doped molecules. Because PMGI nanofiber was able to maintain its structural integrity after being used as a substratum of mouse ESCs (Additional file 1: Figure S1), we examined holding property of PMGI nanofibers toward doped molecules with different chemical properties, which were integrated into the nanofiber through electrospinning, as depicted in the diagram in Figure 1. To this end, 2 small fluorescent molecules, a fluorescein and a porphine, were selected based on their different hydrophobicities: the fluorescein is hydrophilic, whereas the porphine is hydrophobic (Figure 2A). Similarly, 3 peptides were synthesized to have different charges: (1) a peptide composed of negatively charged amino acids, (2) a peptide composed of positively charged amino acids, and (3) a peptide composed of uncharged hydrophobic amino acids. These peptides contained an additional FITC-labeled lysine peptide at their carboxy terminus for monitoring. The fluorescent molecule-doped nanofibers were soaked in water and then spread over a polystyrene dish via repeated pipeting, by which approximately 40 % of the dish surface was covered with the nanofibers (Additional file 2: Figure S2). The coated dishes were incubated at 37°C in a PBS solution for 20 d. During the incubation period, the fluorescence intensity of each nanofiber substratum was measured every 5 day and plotted against its own initial intensity as a percentage. A gradual decrease in fluorescence intensity, which indicates release of the fluorescent molecules, was observed in the nanofiber substrata containing fluorescein or charged peptides composed of lysines or glutamic acids, whereas such a decrease was minimal in the nanofiber substratum containing the porphine or leucine peptide (Figure 2B,C). However, the gradual decreases disappeared after 15 d, resulting in higher than 90 % of the initial fluorescence remaining up to 20 d, regardless of the chemical property of the doped molecules. These results indicate that the doped molecules remained in the PMGI nanofibers irrespective of their chemical properties.

### Doping of gold nanoparticles

**Figure 3:**
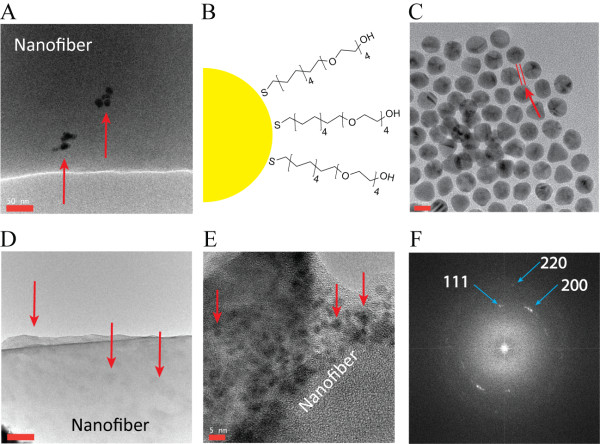
**PEG-labeled AuNPs localized on the surface of the nanofiber.** A few plain AuNPs localized sporadically on the nanofiber surface through electrospinning **(a)**. To enhance stability and surface localization, AuNPs were labeled with the PEG-based molecules **(b)**, by which a 1-nm-thick layer formed (distance between the 2 red lines) on the surface of the AuNPs **(c)**. The PEG-labeled AuNPs mainly localized on the surface of the nanofiber through electrospinning **(d, e)**. The particles shown on the nanofibers were confirmed as gold via FFT analysis **(f)**. The red arrows indicate AuNPs. Scale bars in each figure: A = 50 nm, C = 10 nm, D = 100 nm, and E = 5 nm.

Synthetic substrata for human PSCs is required to have a large amount of cell-adhesive molecules because of the low adhesion of human PSCs. To achieve this, we used a gold nanoparticle (AuNP) because its large surface area enables high-density labeling of functional molecules with low cytotoxicity [42,43]. In addition, AuNPs have been successfully encapsulated into nanofibers through electrospinning [44,45]. The 15-nm unconjugated AuNPs, which were stabilized in citrate buffer, were able to dope the PMGI nanofibers through electrospinning, but they rarely localized on the surface at 1 nM concentration (Figure 3A). We hypothesized that aggregation of the AuNPs might be the reason. To prevent aggregation, the AuNPs were labeled with a PEG-based molecule (PEG), as depicted in the diagram (Figure 3B), and a 1-nm thick layer was formed outside the AuNPs after PEG-labeling (Figure 3C). Higher resolution TEM images supported the formation of PEG layers (Additional file 3: Figure S3). As expected, the surface localization of PEG-labeled AuNPs increased significantly (Figure 3D). A high-resolution TEM image of the nanofiber ensured that the large surface area of the nanofiber was covered with PEG-labeled AuNPs (Figure 3E). The particles on the nanofiber surface were confirmed as Au via an FFT analysis (Figure 3F). Alternatively, increase of the AuNP concentration (≅ 50 nM) enabled surface localization of AuNPs (Additional file 4: Figure S4), but it inhibited attachment of PMGI nanofiber to the dish surface (data not shown). The surface localization of the PEG-labeled AuNPs also changed the hydrophobicity of the nanofibers: the contact angle of the water drop placed on the nanofibers containing the PEG-labeled AuNPs decreased owing to the increased absorption of water (Additional file 5: Figure S5). Thus, AuNPs can dope PMGI nanofibers through electrospinning and their localization on the surface can be controlled.

### Molecular recognition of a doped peptide

**Figure 4:**
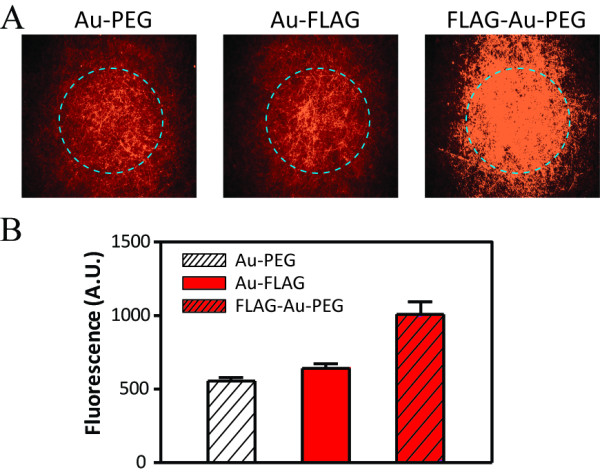
**An anti-FLAG antibody recognized the FLAG peptide in the nanofibers.** Each of the nanofiber substratum containing PEG-AuNPs, FLAG-AuNPs, or AuNPs co-labeled with PEG and FLAG was incubated with an Alexa 555-conjugated anti-FLAG antibody. Fluorescence intensities inside the dashed circles were quantified using a fluorescence microscope at ×40 resolution **(a)**. The mean fluorescence intensity of each nanofiber substratum was calculated by measuring the fluorescence intensities of 5 different areas of each nanofiber substratum **(b)**. The fluorescence intensities indicate the amount of the recognized FLAG peptides in the nanofibers by the fluorescence-labeled anti-FLAG antibody. Error bars: SEM, n = 5.

Next, we investigated whether a peptide can be localized on the nanofiber surface using the PEG-labeled AuNP and electrospinning. The surface localization of the peptide was confirmed via molecular recognition by an antibody. To this end, AuNPs were labeled with the 3X FLAG peptide, which is widely used as a tagging sequence, through a MHDA linker, with or without PEG as depicted in the diagram (Additional file 6: Figure S6). Labeling of the linker and peptide was confirmed by measuring the size of the AuNPs using a Zetasizer (Additional file 7: Figure S7). After doping the FLAG-labeled AuNPs, the nanofibers were incubated with an anti-FLAG antibody labeled with Alexa 555. The molecular recognition of the FLAG peptides by the antibody was quantified by measuring fluorescence intensities of the nanofiber substrata. The AuNP labeled only with FLAG peptide failed to be recognized by the antibody, which was consistent with the rare surface localization on plain AuNPs, whereas a much higher amount of the FLAG peptide labeled the AuNPs was recognized when PEG was present (Figure 4A). The mean fluorescence intensity of 5 different areas in each nanofiber substratum showed surface localization of the FLAG peptide as recognized by the anti-FLAG antibody (Figure 4B).

### Doping with cRGD peptide improved affinity of the nanofiber

**Figure 5:**
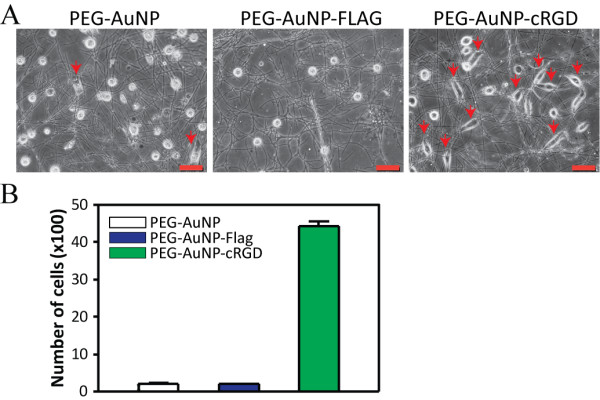
**Enhanced adhesion of HeLa cells to the cRGD-doped nanofiber substratum.** AuNPs labeled with cRGD and PEG were used to dope the PMGI nanofibers through electrospinning, with which a synthetic substratum was fabricated. The same number of HeLa cells were seeded and incubated for 6 h over the cRGD-doped nanofiber substrata and other nanofiber substrata containing plain AuNPs or FLAG-labeled AuNPs. The red arrows indicate attached and spread HeLa cells **(a)**. After washing the cells with PBS, the remaining cells were collected from the 3 dishes of each substratum via trypsinization for quantification **(b)**. Scale bar: 50 μm. Error bars: SEM, n = 3.

Based on the FLAG peptide doping, a cRGD peptide was grafted into the PMGI nanofiber through electrospinning, with which we constructed a nanofiber substratum to examine whether the cRGD-doped nanofiber might have enhanced affinity for binding integrins expressed on the attached cells. Synthetic substrata composed of nanofibers containing PEG-AuNPs or FLAG-labeled AuNPs were fabricated as controls to monitor the effect of PEG or the non-adhesive peptide (FLAG) on cell adhesion, respectively. HeLa cells (500 cells/cm^2^) were spread over each substratum and incubated for 6 h. The cells grown on the nanofiber substratum containing cRGD revealed enhanced attachment and spreading, as determined by the formation of its unique shape indicated by the red arrows, whereas there was no cell or only a limited number of cells with such attachment and spreading on the substrata containing FLAG-AuNPs or PEG-AuNPs, respectively (Figure 5A). The strongly attached cells were collected from 3 dishes of each nanofiber substratum via trypsinization. The mean number of collected cells from the cRGD-containing substrata was significantly higher than that from the other 2 substrata (Figure 5B), which indicates that the adhesive function of cRGD can be grafted upon nanofibers through electrospinning.

### A functionalized nanofiber substratum for cardiomyocyte differentiation

**Figure 6:**
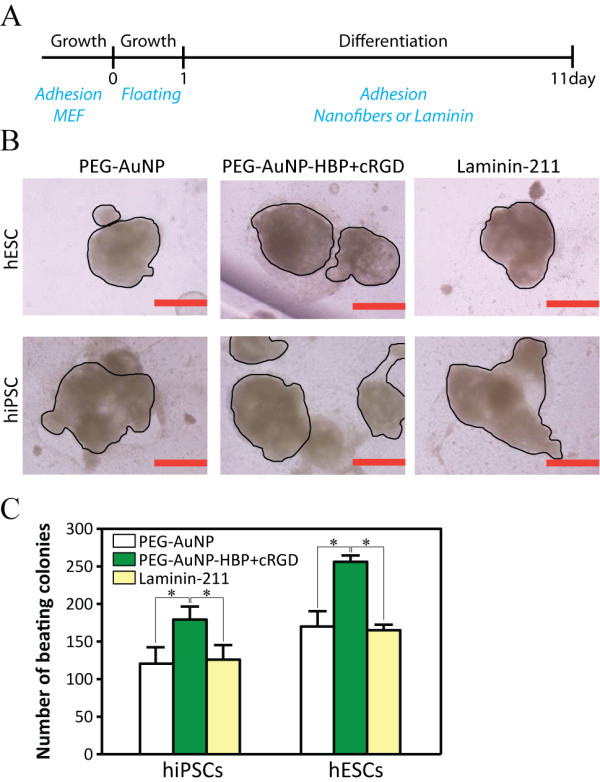
**The functionalized nanofiber substratum potentiates cardiomyocyte differentiation.** Human PSCs were cultured over the MEF. To induce cardiomyocyte differentiation, EBs were formed by culturing the PSCs over ultra-low-attachment culture dishes. The same number of EBs were spread and differentiated for 10 d over each of the substratum as depicted in the diagram **(a)**. Laminin-211 indicates laminin-211-coated dishes, PEG-AuNP indicates nanofiber substrata containing the PEG-AuNPs, and PEG-AuNP-HBP + cRGD indicates nanofiber substrata containing PEG-AuNPs functionalized with HBP and cRGD. After 10 d of differentiation, cell clumps showing spontaneous contractility appeared. A single cardiomyocyte colony was defined as a cell clump showing synchronized beating (black outlined), irrespective of its size **(b)**. Cardiomyocyte colonies generated in 3 dishes of each substratum were counted for quantification **(c)**. Scale bars: 500 μm. Error bars: SEM, n = 3. The asterisks indicate statistical significance (*P* < 0.01) calculated using the Student *t* test. The results shown represent 3 independent experiments. PSC: pluripotent stem cell, MEF: mouse embryonic fibroblast, EB: embryoid body, Growth: growth medium, and Differentiation: differentiation medium.

Similar to the cRGD-doped nanofiber substratum, the nanofibers containing AuNPs labeled with 2 adhesive peptides, a cRGD peptide and a HBP, were used to fabricate a substratum for cardiomyocyte differentiation of human PSCs. The functionality of the nanofiber substratum was compared with that of laminin-211, a proven substratum for human PSCs. A nanofiber substratum containing PEG-AuNPs was also used to evaluate the structural functionality of the nanofiber to cardiomyocyte differentiation. Human ESCs (khES1) and iPSCs (201B7), which were maintained over MEFs in the growth medium, became embryoid bodies (EBs) via suspension culture. The same number of the EBs were spread over 3 dishes of each substratum and then induced to differentiate into cardiomyocytes using the differentiation medium (Figure 6A). After 10 d, spontaneous contractile colonies began to appear. The effect of each substratum on cardiomyocyte differentiation was evaluated based on the mean number of beating colonies developed in each substratum. A beating colony was defined as a clump of cells showing synchronized contractility, irrespective of its size (Figure 6B, outlined). The mean number of beating colonies was statistically higher in the nanofiber substratum containing cRGD and HBP than in the laminin-211 substratum or in the nanofiber substratum containing PEG-AuNP (*P* < 0.01). Unexpectedly, the mean colony number from the nanofiber substrata containing PEG-AuNPs was comparable with that from the laminin-coated substrata (Figure 6C). Thus, the PMGI nanofiber substratum functionalized with the adhesive peptides through electrospinning enhanced cardiomyocyte differentiation of human PSCs even better than did laminin-211.

## Discussion

Nanofibers are proven substrata for culturing somatic cells, but their application to PSCs has recently been investigated [46]. Herein, we introduced an application of the nanofiber substratum to cardiomyocyte differentiation of human PSCs. Usually, surface modifications, such as amines [21], carboxylic acids [47], and alkanethiols [23], are required for grafting cell adhesive molecules on synthetic substrata. However, our results indicate that co-electrospinning can also be used as a method to graft cell adhesive peptides when PMGI nanofibers and PEG-labeled AuNPs are used. The rigid structure of the PMGI nanofibers enabled incorporation of the AuNPs, whereas the PEG-labeled AuNPs delivered a large amount of the peptides onto the nanofiber surface. Such high-density localization of the functional peptides on the nanofibers fulfills the biophysical and biochemical environmental cues required for PSC culture [23,48]. Embedding of AuNPs into nanofibers has been introduced to add mechanical functions to the nanofibers: AuNPs were incorporated in silica nanofibers and in TiO_2_ nanofibers through electrospinning for the wavelength-dependent photoelectric response and for enhancing lithium-ion diffusion and charge transfer, respectively [44,45]. Unlike the previous reports, we used AuNPs to add biological functions to the nanofibers. The proof of concept of such “AuNP doping” through electrospinning was confirmed by recognition of the FLAG and cRGD peptides, which were localized on the nanofiber surface via the AuNP, by the anti-FLAG antibody and RGD receptors expressed on HeLa cells, respectively. HeLa cells are known to follow 3 events during cell adhesion: initial attachment, spreading, and elaborate interaction with the substratum using their 3 types of RGD receptors [49]. Therefore, the strong attachment and spreading of HeLa cells on the cRGD-doped nanofiber substratum indirectly indicates recognition of cRGD by the RGD receptors on the HeLa cells. Similarly, a nanofiber substratum functionalized with cRGD and HBP enhanced cardiomyocyte differentiation of the human PSCs. These 2 peptides have been known to potentiate self-renewal of human PSCs [23], and we found that the combination of these 2 peptides also potentiated cardiomyocyte differentiation better than did laminin-211 (Figure 6). Nevertheless, these 2 peptides may not be the best functional materials for potentiating cardiomyocyte differentiation, considering the increase of cell clumps that didn't beat were observed simultaneously in the peptide-doped nanofiber substratum (data not shown). These 2 peptides would be rather conventional substrata for diverse cell types combined with specific culture medium. Screening of peptides and small molecules that relatively specific to cardiomyocyte differentiation will be conducted using this nanofiber method. It is interesting that nanofibers containing PEG-AuNPs potentiated cadiomyocyte differentiation as much as the laminin-211 did, which was an unexpected result because cardiomyocyte differentiation of human PSCs was erratic and poor over the plain culture dishes that had neither biophysical nor biochemical environmental cues from the extracellular matrix; we observed significant decrease of beating colony formation with high batch-to-batch variation when plain dishes were used instead of laminin-211 coated dishes (data not shown). We infer that it might be caused by preference of cardiac cells for the nanofibrous structure, even in the absence of the biochemical cues as reported [50].

The method described here has attractive points in terms of cost, convenience, and applicability: the PMGI nanofiber is cheap, and functionalization is achieved through simple co-electrospinning and can be applied to examine concentration-dependent effects and combinatorial effects of different functional molecules to the cells. The produced functionalized nanofibers can be applied to lithographic patterning of the nanofibers as described [35] for more precisely controlled differentiation of PSCs and also used to develop a cell type-specific substratum for the cells having unique characteristics, such as motor neurons and hepatocytes, which are difficult to maintain or obtain in vitro using a conventional substratum.

## Conclusions

Our findings indicate that PMGI nanofibers can integrate small molecules, peptides, and functionalized AuNPs through electrospinning. Using this technique, we were able to fabricate synthetic nanofiber substrata that enhanced HeLa cell adhesion or potentiated cardiomyocyte differentiation of human PSCs.

We believe that these results would serve as a foundation to fabricate diverse cell type-specific substrata.

## Competing interests

The authors declare that they have no competing interests.

## Authors’ contributions

DJ, SP, JL MI, JB, QY, LL, and SK performed the research; DJ, YC, KL, and NN analyzed the data and wrote the manuscript. All the authors read and approved the final manuscript.

## Supplementary Material

Additional file 1 **Figure S1. SEM images of PMGI nanofibers.** Nanofibers maintained their structure. (A) Structure of newly produced PMGI nanofibers. (B) A mouse ESC colony cultured over PMGI nanofibers for 1 week was observed. Click here for file

Additional file 2 **Figure S2. The surface of a polystyrene dish can be coated with PMGI nanofibers by pipeting.** Rhodamine 6B (10-μM final concentration) was premixed in 13 % PMGI solution and then incorporated into the nanofibers via electrospinning. A polystyrene dish (10 cm) was coated with the Rhodamine-doped nanofibers by pipeting the nanofibers repeatedly against the surface. The fluorescence image indicates that Rhodamine remains in the nanofiber under PBS, which was monitored with a fluorescence confocal microscope. Scale bars: 50 μm. Click here for file

Additional file 3 **Figure S3. TEM images of PEG-labeled AuNPs.** High resolution TEM images showed formation of PEG layer on the surface of AuNPs. Red arrows indicate the PEG layer. Scale bars: A = 10 nm and B = 5 nm. Click here for file

Additional file 4 **Figure S4. TEM images of PMGI nanofiber containing higher number of AuNPs.** Plain AuNPs (≅ 50 nM) were integrated into PMGI nanofiber through co-electrospinning with which a TEM image was taken. Scale bars: A = 0.5 μm and B = 100 nm. Click here for file

Additional file 5 **Figure S5. Contact angle measurements.** The high-density PMGI nanofibers were fixed on a solid polydimethylsiloxane (PDMS) gel using adhesive silicon on which a drop of Mili-Q water was placed on the nanofiber meshes to measure its contact angle. Surface localization of the PEG-labeled AuNP made the nanofiber substratum hydrophilic, resulting in a decreased contact angle of the water drop. Au and Au-PEG indicate plain gold nanoparticle and polyethylene glycol-labeled gold nanoparticle, respectively. Click here for file

Additional file 6 **Figure S6. Labeling process of a AuNP with functional peptides.** The surface of a 15-nm AuNP was labeled with 75 % PEG and 25 % MHDA using equal amounts of cRGD and HBP covalently conjugated through EDC/NHS coupling. EDC: 1-ethyl-3-(3-dimethylaminopropyl)carbodiimide, NHS: *N*-hydroxysuccinimide, and MHDA: 16-mercaptohexadecanoic acid. Click here for file

Additional file 7 **Figure S7. Size measurement of a functionalized AuNP.**The size of an AuNP labeled with the 3X FLAG peptide (NH_2_-MDYKDHDGDYKDHDIDYKDDDDK-OH) through MHDA was measured. The surface of a 15-nm AuNP (A) was covered with 75 % PEG and 25 % MHDA (B), and the FLAG peptide was conjugated at the MHDA (C). The growing size of the AuNP after each labeling process was measured using a Zetasizer. The actual size of the functionalized FLAG-AuNP almost exactly matched the predicted size, which was calculated based on the length of the linker and 3X FLAG peptide. The results represent 3 independent measurements. Click here for file
