# Supplementary figures and images for: Incorporation of functionalized gold nanoparticles into nanofibers for enhanced attachment and differentiation of mammalian cells
Source: J Nanobiotechnology. 2012 Jun 11;10:23. doi: 10.1186/1477-3155-10-23 (PMC3431254; doi:10.1186/1477-3155-10-23)

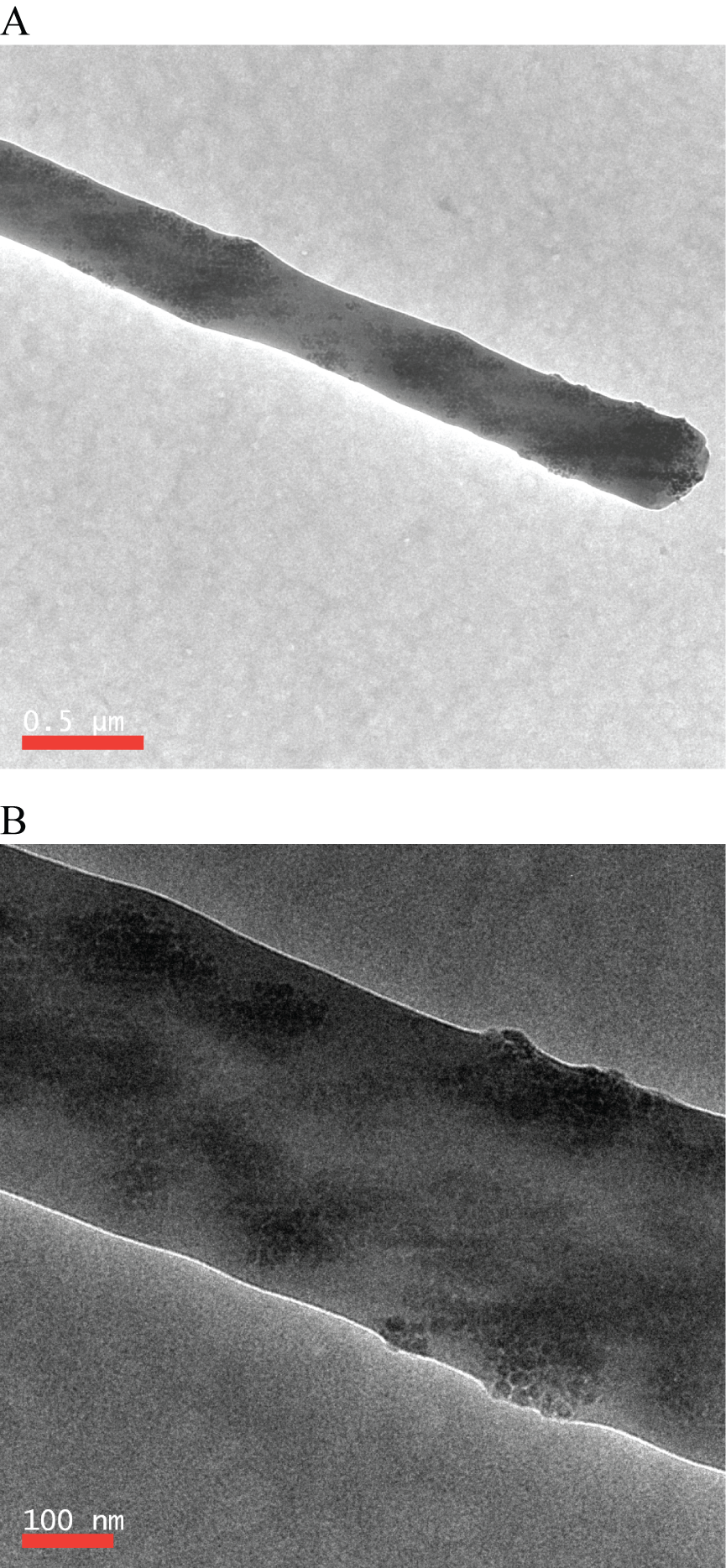

Supplement: Additional file 1 — Figure S1. SEM images of PMGI nanofibers. Nanofibers maintained their structure. (A) Structure of newly produced PMGI nanofibers. (B) A mouse ESC colony cultured over PMGI nanofibers for 1 week was observed. [file 1477-3155-10-23-S1.tiff]

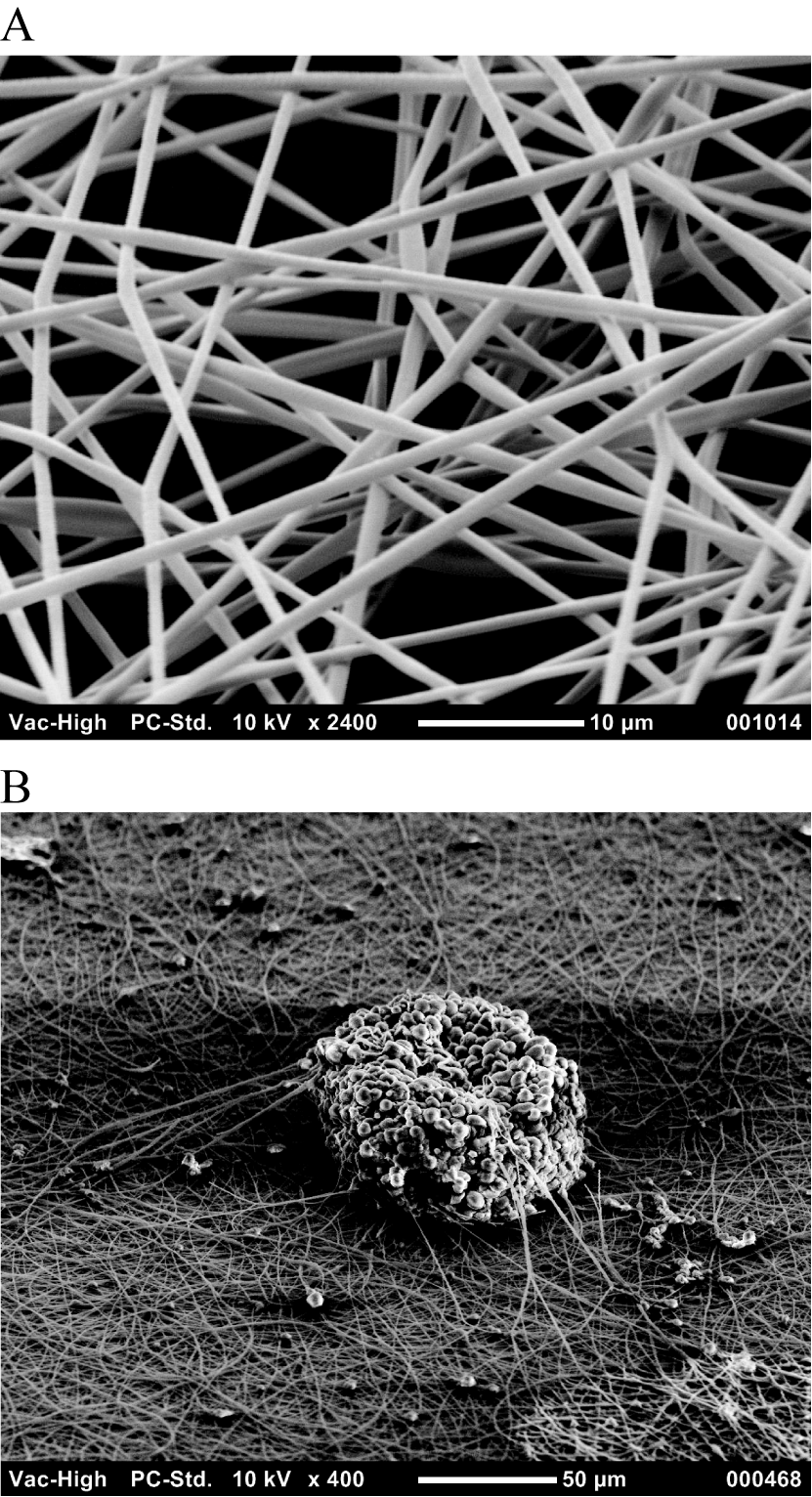

Supplement: Additional file 2 — Figure S2. The surface of a polystyrene dish can be coated with PMGI nanofibers by pipeting. Rhodamine 6B (10-μM final concentration) was premixed in 13 % PMGI solution and then incorporated into the nanofibers via electrospinning. A polystyrene dish (10 cm) was coated with the Rhodamine-doped nanofibers by pipeting the nanofibers repeatedly against the surface. The fluorescence image indicates that Rhodamine remains in the nanofiber under PBS, which was monitored with a fluorescence confocal microscope. Scale bars: 50 μm. [file 1477-3155-10-23-S2.tiff]

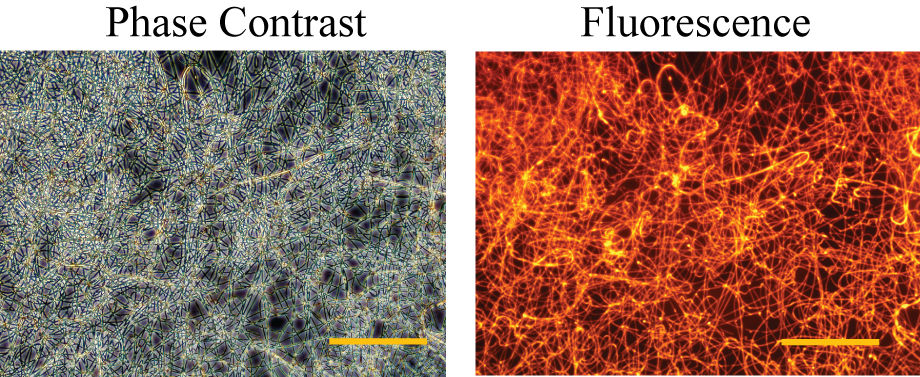

Supplement: Additional file 3 — Figure S3. TEM images of PEG-labeled AuNPs. High resolution TEM images showed formation of PEG layer on the surface of AuNPs. Red arrows indicate the PEG layer. Scale bars: A = 10 nm and B = 5 nm. [file 1477-3155-10-23-S3.tiff]

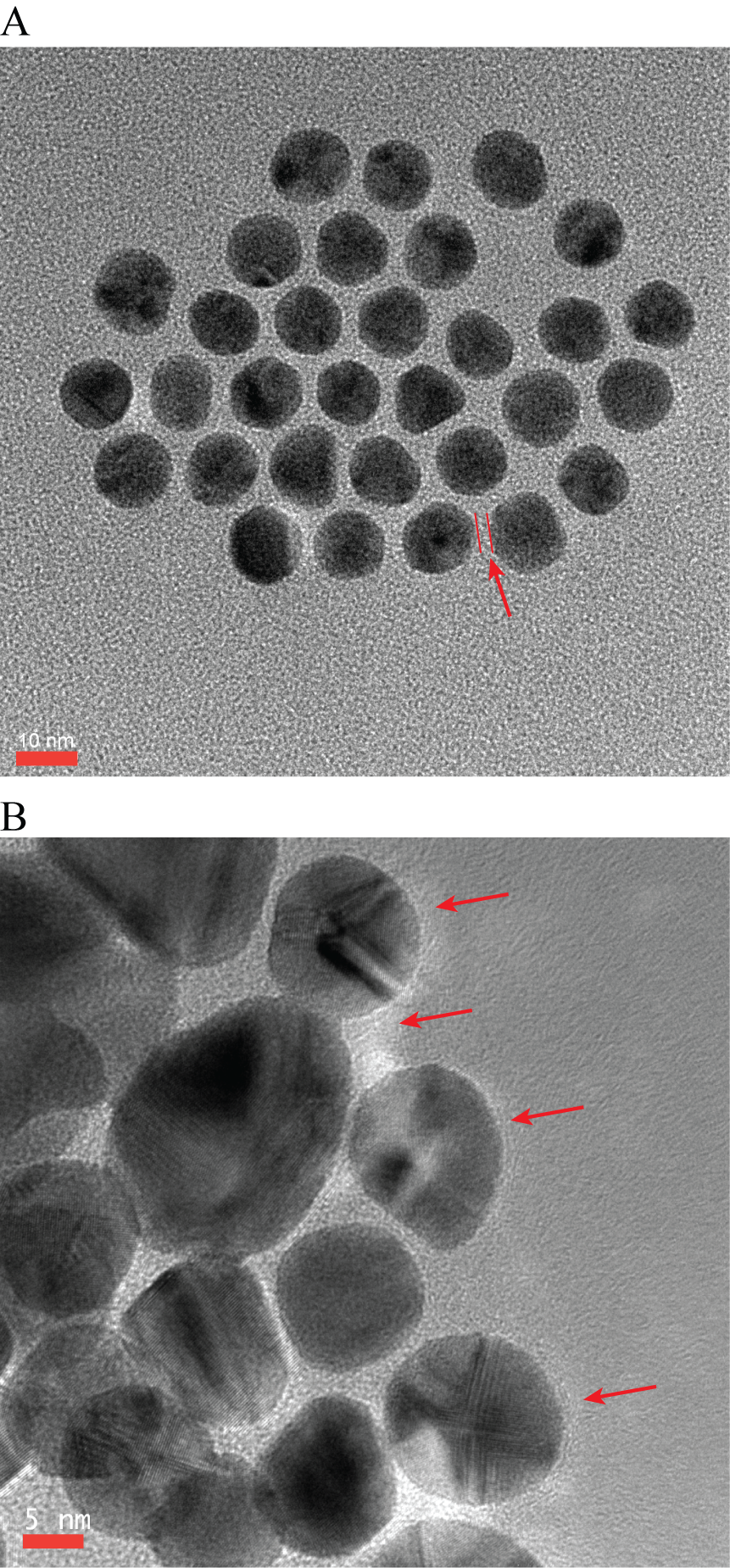

Supplement: Additional file 4 — Figure S4. TEM images of PMGI nanofiber containing higher number of AuNPs. Plain AuNPs (≅ 50 nM) were integrated into PMGI nanofiber through co-electrospinning with which a TEM image was taken. Scale bars: A = 0.5 μm and B = 100 nm. [file 1477-3155-10-23-S4.tiff]

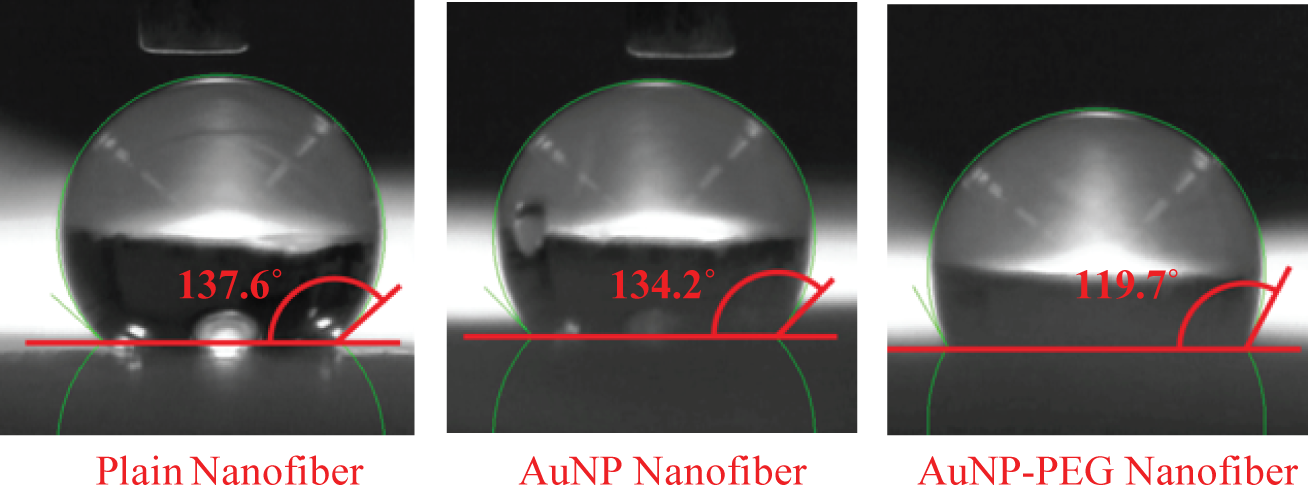

Supplement: Additional file 5 — Figure S5. Contact angle measurements. The high-density PMGI nanofibers were fixed on a solid polydimethylsiloxane (PDMS) gel using adhesive silicon on which a drop of Mili-Q water was placed on the nanofiber meshes to measure its contact angle. Surface localization of the PEG-labeled AuNP made the nanofiber substratum hydrophilic, resulting in a decreased contact angle of the water drop. Au and Au-PEG indicate plain gold nanoparticle and polyethylene glycol-labeled gold nanoparticle, respectively. [file 1477-3155-10-23-S5.tiff]

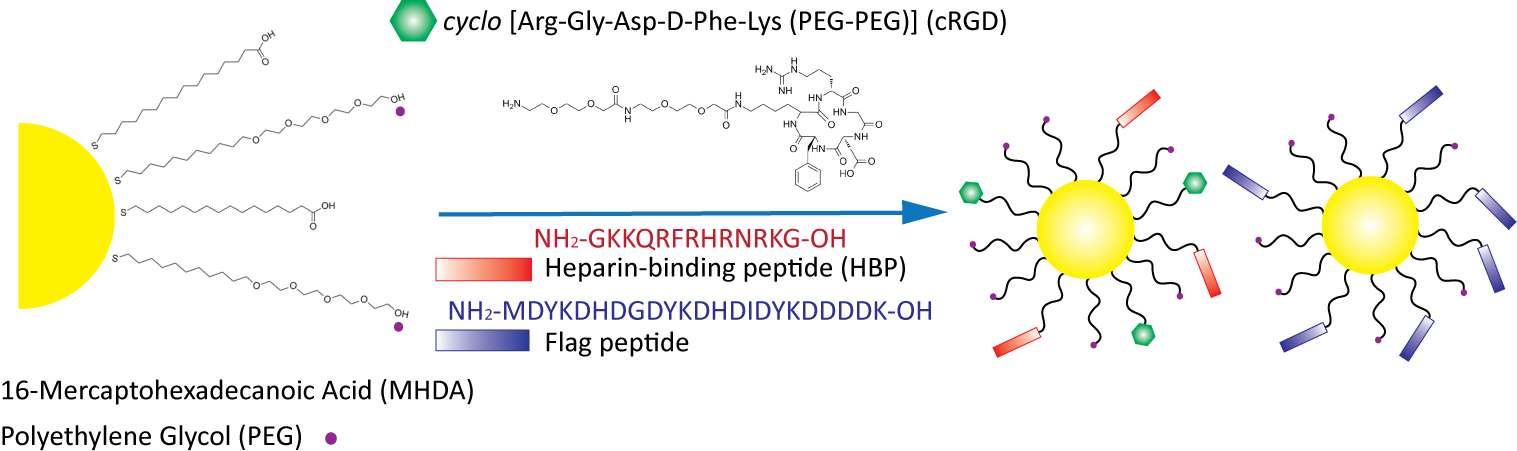

Supplement: Additional file 6 — Figure S6. Labeling process of a AuNP with functional peptides. The surface of a 15-nm AuNP was labeled with 75 % PEG and 25 % MHDA using equal amounts of cRGD and HBP covalently conjugated through EDC/NHS coupling. EDC: 1-ethyl-3-(3-dimethylaminopropyl)carbodiimide, NHS: N-hydroxysuccinimide, and MHDA: 16-mercaptohexadecanoic acid. [file 1477-3155-10-23-S6.tiff]

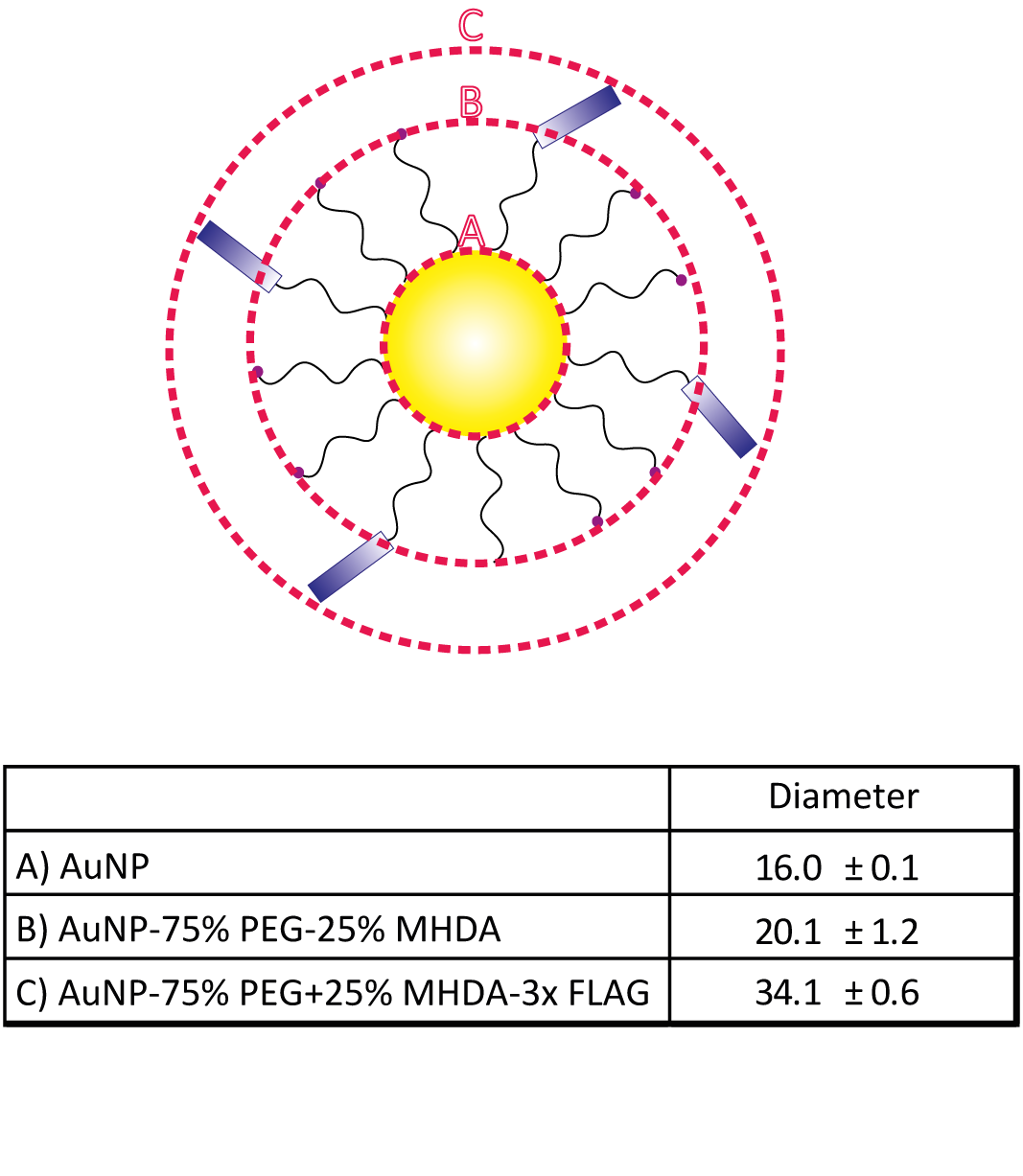

Supplement: Additional file 7 — Figure S7. Size measurement of a functionalized AuNP.The size of an AuNP labeled with the 3X FLAG peptide (NH2-MDYKDHDGDYKDHDIDYKDDDDK-OH) through MHDA was measured. The surface of a 15-nm AuNP (A) was covered with 75 % PEG and 25 % MHDA (B), and the FLAG peptide was conjugated at the MHDA (C). The growing size of the AuNP after each labeling process was measured using a Zetasizer. The actual size of the functionalized FLAG-AuNP almost exactly matched the predicted size, which was calculated based on the length of the linker and 3X FLAG peptide. The results represent 3 independent measurements. [file 1477-3155-10-23-S7.tiff]
